# Delivery of miR-34a by chitosan/PLGA nanoplexes for the anticancer treatment of multiple myeloma

**DOI:** 10.1038/srep17579

**Published:** 2015-12-01

**Authors:** Donato Cosco, Felisa Cilurzo, Jessica Maiuolo, Cinzia Federico, Maria Teresa Di Martino, Maria Chiara Cristiano, Pierfrancesco Tassone, Massimo Fresta, Donatella Paolino

**Affiliations:** 1 Department of Health Sciences, University “Magna Græcia” of Catanzaro, Campus Universitario “S. Venuta”, Viale S. Venuta, Germaneto, I-88100 Catanzaro, Italy; 2 Department of Experimental and Clinical Medicine, University “Magna Græcia” of Catanzaro, Campus Universitario “S. Venuta”, Viale S. Venuta, Germaneto, I-88100 Catanzaro, Italy; 3 Sbarro Institute for Cancer Research and Molecular Medicine, Center for Biotechnology, College of Science and Technology, Temple University, Philadelphia, PA, US; 4 IRC FSH- Interregional Research Center for Food Safety & Health, University of Catanzaro “Magna Græcia”, Campus Universitario “S. Venuta” - Building of BioSciences, Viale S. Venuta, I-88100 Germaneto; Catanzaro, Italy

## Abstract

The encapsulation of miR-34a into chitosan/PLGA nanoparticles in order to obtain nanoplexes useful for the modulation of the biopharmaceutical features of the active compound was studied. The nanoplexes were obtained through nanoprecipitation and were characterized by a mean diameter of ~160 nm, a good size distribution and a positive surface charge. The structure of the nanoparticles allowed a high level of entrapment efficiency of the miR-34a and provided protection of the genetic material from the effects of RNase. A high degree of transfection efficiency of the nanoplexes and a significant *in vitro* antitumor effect against multiple myeloma cells was demonstrated. The therapeutic properties of the nanoplexes were evaluated *in vivo* against human multiple myeloma xenografts in NOD-SCID mice. The systemic injection of miR-34a mimic-loaded nanoparticles significantly inhibited tumor growth and translated into improved survival of the laboratory mice. RT-PCR analysis carried out on retrieved tumors demonstrated the presence of a high concentration of miR-34a mimics. The integrity of the nanoplexes remained intact and no organ toxicity was observed in treated animals.

The genetic deregulation of specific genes characterizes a wide range of serious acquired and inherited diseases, including cancer. Cancer research is focused on the development of alternative approaches to traditional therapies, that include not only the identification of new antitumor agents but also their specific delivery to cancerous tissues through the use of innovative nanodevices^1^,^2^,^3^. In particular, the discovery of new molecular pathways involved in cancer diseases, such as the role of interference RNA, has allowed the identification of new molecular targets that can be employed for the efficacious treatment of cancer, so great interest is focused on their potential therapeutic use as ‘new drugs’, as in the case of medium interference RNA (miRNA)^4^,^5^.

The therapeutic use of genetic material is one of the most recent and innovative approaches in the treatment of cancer and is aimed at the introduction and/or replacement of genetic material in specific tumor target cells. The deep deregulation of microRNAs (miRNAs) plays a relevant role in the pathogenesis of several human malignancies, including multiple myeloma (MM), a plasma cell dyscrasia characterized by the clonal accumulation of monotypic paraprotein-secreting cells in the bone marrow^6^. Among the types of deregulated miRNA present in MM, miR-34a, a well-known tumor suppressor miRNA, has been shown to be of potential value in the treatment of MM^7^,^8^. The transcription of miR-34a is induced by the tumor suppressor gene p53, which is disactivated in most cancers. The mechanism of miR-34a seems to be related to the direct modulation of downstream targets Bcl-2, Notch 1 and CDK6^9^.

A crucial point for the potential clinical translation of an miRNA therapeutic approach is strongly related to the use of a suitable device which should be able to allow an efficient cellular uptake along with preservation of the pharmacological activity of the encapsulated compound, through its protection from the rapid enzymatic-based degradation processes. In fact, the limiting factors for the intracellular uptake of a naked gene are its hydrophilicity, negative charge, high molecular weight, elevated hydrodynamic size and the rapid degradation it undergoes caused by serum endonucleases and the reticuloendothelial system (RES)^10^.

Therefore, the development of safe and efficient gene delivery systems is an exciting challenge in the field of pharmaceutical research, in the attempt to provide potential platforms to be used for genetic therapy^11^,^12^. An interesting approach is based on the use of polymeric nanoparticles, which have been demonstrated to be suitable for a wide range of applications for the delivery of a number of substances; these nanoparticles have the aim of improving the biopharmaceutical features of the encapsulated drug as well as its sustained release^13^,^14^. Our research team recently developed a nanoparticle device made up of chitosan, poloxamer 188 and PLGA, that was able to efficiently encapsulate and deliver genetic material because it showed physicochemical parameters suitable for making it a possible candidate for systemic administration, low *in vitro* cytotoxicity and significant transfection features^15^.

These nanodevices have the potential to suitably deliver miRNAs through the modulation of their biopharmaceutical properties and their antitumor activity, while the encapsulation of miR-34a in chitosan/PLGA nanoparticles could provide a nanomedicine for the conceivable treatment of multiple myeloma. This is why this investigation was focused on the *in vitro* and *in vivo* evaluation of the plausible application of miR-34a-chitosan/PLGA nanoplexes. This was done by testing the following: the protection of the entrapped miR-34a from degradation by ribonuclease; the *in vitro* antiproliferative effect on multiple myeloma cells; the *in vivo* anticancer activity of miR-34a-chitosan/PLGA nanoplexes in murine xenograft models of human multiple myeloma disease; the expression of miR-34a in retrieved tumors and the direct modulation of its Bcl-2 and CDK6 downstream targets.

## Results

### Physico-chemical characterization of nanoplexes

**Figure 1:**
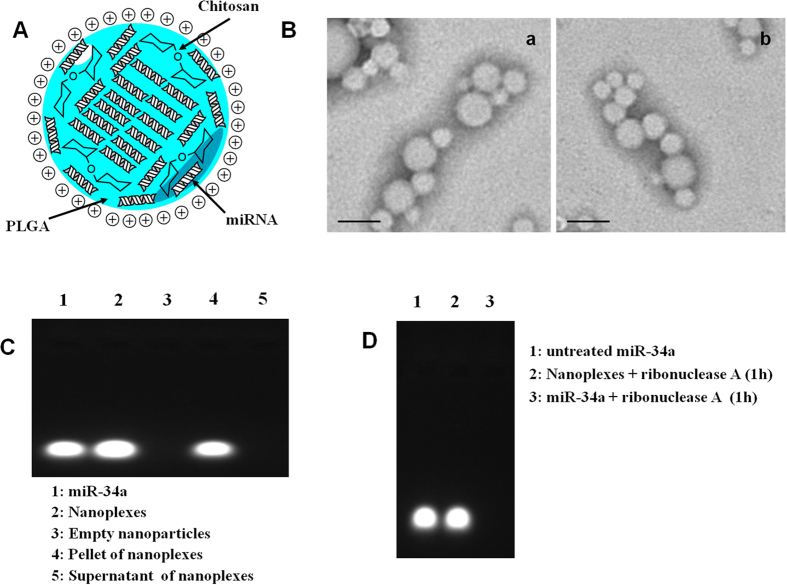
Physico-chemical characterization of nanoplexes made up of miR-34a-loaded chitosan/PLGA nanospheres. Panel A: schematic representation; panel B: TEM micrographs of empty nanosystems (**a**) and of nanoplexes (**b**), bar = 200 nm. The morphology of the nanosystems was evaluated using a transmission electron microscope (Philips, Eindhoven, The Netherlands). Samples were analyzed at 100 kV. A drop of the sample was deposited on a copper screen coated with carbon. The sample was dried and then contrasted with uranyl acetate for 2 minutes and then washed with distilled water. Panel C: gel retardation assay. Nanoplexes were centrifuged and the pellet destroyed with an aqueous solution of SDS (1.0% w/v) in order to assay the presence of miR-34a. To each 10 μl sample, 2 μl of 6 × loading dye were added totaling a final volume of 12 μl. The complexes were then loaded onto 1% agarose gel in Tris-acetate-EDTA (TAE) buffer containing 0.5 μg/μl of ethidium bromide. Electrophoresis was run at 120 V for 30 min. The miR-34a bands were visualized using a UV-20 transilluminator (Hoefer Pharmacia Biotech Inc., San Francisco, CA, USA). Naked miR-34a and blank nanoparticles were used as controls. Panel D: RNase A protection assay.

Poloxamer 188 was used as the surfactant for the preparation of the nanoplexes due to its ability to preserve and repair the cell membrane, thus increasing the structural stability of the cell^16^. The use of PLGA nanoparticles in gene delivery can be limited by the negative charge of the polymer, which prohibits a fine interaction with the negative charge of the phosphate group of nucleic acids. This is why chitosan was chosen as a condensing agent, thanks to its cationic nature which is useful for establishing electrostatic interaction with polyanions (Fig. 1 panel A). This system was already characterized and its ability to retain genetic material plasmids and to allow significant cell transfection was demonstrated^15^.

The encapsulation of miR-34a required a novel characterization of the investigated nanoplexes in order to evaluate the influence of this macromolecule on the physico-chemical features of the colloidal nanocarrier. The mean size, polydisperity index (PI) and zeta potential of empty and miR-34a-loaded nanoparticles were evaluated by DLS analysis. Chitosan/PLGA nanospheres evidenced a mean diameter of ~170 nm, a PI of ~0.2 and a surface charge of 45 mV, deriving from the protonation of the amino-residues of chitosan. The positive charge of the matrix is useful for promoting the retention of genetic material inside the particles. The encapsulation of miR-34a induced a proportional decrease of both the mean diameter and the Zeta potential of nanoplexes as a function of the drug concentration (Table 1). Namely, the nanoplexes showed a mean size of ~150 nm and a surface charge of ~25 mV when 500 μg of miR-34a were used in their preparation. This result was due to the ability of the nanoplexes to package the poly-anionic miR-34a by way of an electrostatic interaction which comes about between the negatively-charged phosphate residue of the nucleic acid and the positively-charged amino-residues of chitosan. The encapsulation of miR-34a did not modify the morphology of the nanoplexes, which were characterized by a smooth spherical shape (Fig. 1 panel B) as evidenced by TEM analysis.

The entrapment efficiency (EE) and the yield (EY) are fundamental parameters to be evaluated, particularly in the case of the delivery of biologically active macromolecules such as miR-34a; in fact, an effective entrapment and a suitable loading yield can positively influence the *in vivo* therapeutic response, besides heighten the possibility of its potential clinical application. Different amounts of miR-34a (ranging from 100 to 500 μg) were added to the aqueous phase (5 ml) during the preparation of the nanoplexes, thus obtaining high EE values. In particular, the use of 300 μg of miR-34a favored a drug load of ~85%, a satisfactory value for their potential *in vivo* application. The high loading efficiency and the decrease of Zeta potential when miR-34a was added to the nanoplexes confirmed the capacity of chitosan/PLGA nanoplexes to interact with genetic material.

The ability of the nanoparticles to interact and retain miR-34a and the effective encapsulation of this compound were qualitatively investigated by agarose gel electrophoresis. Panel C in Fig. 1 shows the migration of the miR-34a contained in the nanoplexes (lane 2) as compared to that of the free compound (lane 1). The characteristic fluorescent band confirmed that the structure of the nucleic acid was not destabilized during the preparation procedure (see supplementary information). Moreover, this experiment further supported the evidence of the efficient loading of the miR-34a into the nanoplexes (the empty formulation did not show fluorescent bands) (lane 3). In order to confirm the presence of miR-34a inside the nanospheres, they were centrifuged and the pellet was disgregated in order to verify the amount of the entrapped miR-34a (lane 4). This experiment evidenced a great concentration of miR-34a within the nanoplexes, while the amount contained in the supernatant after centrifugation was undetectable (lane 5).

The stability of the nanoplexes is another essential pre-requisite for their potential application as gene delivery systems. The physical stability of empty and miR-34a-loaded nanoplexes was also investigated through multiple light scattering (MLS). The comparison of the stability indexes of the empty nanoplexes and the nanoplexes prepared with 300 μg of miR-34a evidenced no significant alteration of the backscattering and transmission profiles (see supplementary information).

The incubation of miR-34a-loaded nanoplexes or oligofectamine in 60% (v/v) FBS confirmed the greater stability of the polymeric formulation in biological fluids with respect to the commercial formulation. The chitosan/PLGA nanoplexes showed neither a rapid nor a great increase of their mean sizes. Only after 24 h incubation was an increase of the mean sizes greater than 300 nm, thus rendering the nanoplexes no longer suitable for systemic administration (see supplementary information). This phenomenon is related to the cationic nature of nanoplexes which favors their aggregation as a consequence of their interaction with serum components which gives rise to the formation of a protein corona network around them^17^. However, the duration of the serum stability of the miR-34a nanoplexes was sufficient to ensure suitable therapeutic application and tumor localization following their systemic administration.

### Protection of nanoplexes loaded with genetic material

**Figure 2:**
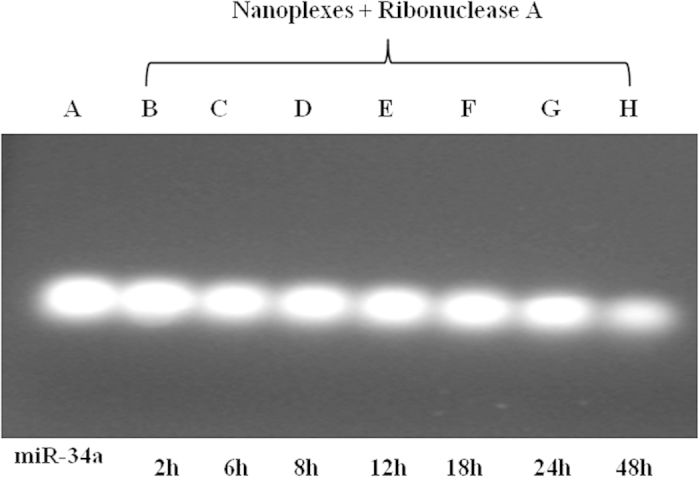
RNase A protection assay of miR-34a-loaded chitosan/PLGA nanospheres as a function of the incubation time. RNase A was added to nanoplexes or naked miRNA and incubated at 37 °C for 1 h. Gel electrophoresis was carried out in 1% (w/v) agarose gel for 40 min at 120 V using TAE buffer. The experiments were carried out as a function of time ranging from 1 h to 48 h.

Genetic material should be protected from degradation on the part of the serum enzymes such as ribonuclease in order to have potential *in vivo* use in nanoplexes. The phosphodiester backbone of RNA is very sensitive to hydrolysis, so naked miR-34a and miR-34a-loaded nanoplexes were incubated with ribonuclease A, in order to investigate the ability of the polymeric nanoplexes to preserve the integrity of the compound. After 1 h incubation, the naked miR-34a was totally degraded by the enzyme (Fig. 1, panel D, lane 3) while the nanoplexes proved able to efficiently protect it (Fig. 1, panel D, lane 2), since no degradation of the miR-34a was evidenced. The same experiment was repeated at different incubation times (from 1 h to 48 h) in order to evaluate the extent of the protection the nanoplexes afforded towards the miR-34a as a function of time. Figure 2 shows the characteristic fluorescent band of miR-34a up to 48 h incubation (lanes B to G), thus evidencing that no degradation of the material occurred. The fluorescent band was really indicative only of the entrapped miR-34a because the release of the miR-34a did not elicit the characteristic fluorescent band due to its rapid degradation carried out by the ribonucleases. This experiment evidenced the high condensing properties the nanoplexes exerted on the genetic material and the slow release of miR-34a that occurs from the nanoplexes. A slight destabilization of the nanoplexes was observed after 48 h incubation, as shown by the weakened fluorescent band (Fig. 2, lane H).

### Evaluation of cytotoxicity and transfection features

The *in vitro* antitumor effect of miR-34a-loaded nanoplexes in terms of both dose- and incubation time-response was investigated by using the MTT-test on SKMM1 and 8226 cells.

Firstly, the extremely low toxicity of empty chitosan/PLGA nanoplexes towards MM cells was demonstrated, which means that any anti-proliferative effect that occurs has to be attributed to the entrapped miR-34a.

**Figure 3:**
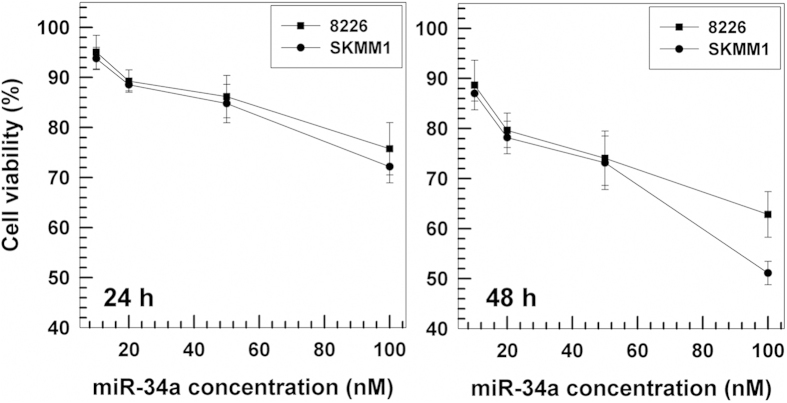
*In vitro* cytotoxicy of miR-34a-loaded chitosan/PLGA nanospheres on MM cells as a function of miRNA concentration and exposition time. Data are expressed as percentages of cellular viability as evaluated by MTT-testing. Results are the mean of four different experiments ± standard deviation.

Then it was observed that a significant decrease in cell viability was brought about by the nanoplexes at the highest miR-34a concentration (0.1 μM) after 24 h incubation, and this finding was confirmed after 48 h (Fig. 3). In particular, at a miRNA concentration of 100 nM, the nanoplexes induced a decrease in cell vitality of 50% and 35% on SKMM1 and 8226 cells, respectively. Significant cytotoxicity was also evident at lower drug concentrations; for instance, a cell viability of 75–80% was observed on both cell lines when 50 nM of miR-34a was used.

**Figure 4:**
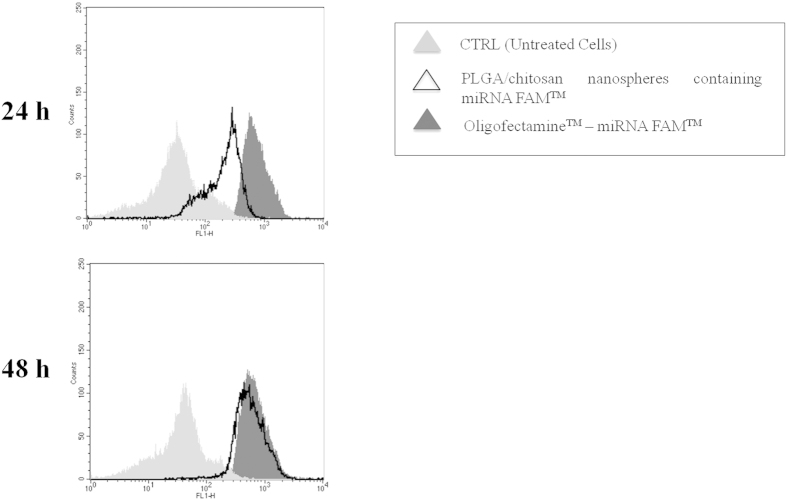
FACS analysis of SKMM1 cells treated for 6 h with miRNA-FAM^TM^-loaded chitosan/PLGA nanospheres or oligofectamine/miRNA-FAM^TM^. MM cells were seeded in 6-well culture dishes (1 × 10^6^ cells/well) in an antibiotic-free medium containing the formulations (3 pmol of FAM^TM^-labeled pre-miR/well). After 6 h incubation, the cells were washed and incubated for 24 and 48 h with fresh medium. Successively, the cells were centrifuged, the obtained supernatant was removed and the pellet was re-suspended in pre-chilled PBS. The cells were then transferred into a 5 ml polystyrene round-bottom tube for subsequent flow-cytometry analysis. The fluorescence intensities of FAM were recorded in the FL1 channel using a FACSCan (Becton Dickinson, USA) flow cytometer. The experiments were performed in triplicate. FL1-H on the X-axis is the fluorescence channel used. Cell counts are reported on the y-axis.

In order to demonstrate that the antitumor effect of nanoplexes is related to their ability to favor the cellular uptake of genetic materials, the transfection efficiency of the nanosystems was investigated through flow cytometry analysis. The transfection experiments were performed by means of miRNA-FAM™ as a fluorescent model compound. Experiments were carried out on SKMM1 cells due to their sensitivity towards the antiproliferative activity of the miR-34a as demonstrated by the *in vitro* viability tests (Fig. 4). The cells were treated with both miRNA-FAM™-loaded nanospheres and with a complex of Oligofectamine^TM^/miRNA-FAM™ (positive control) for 6 h. Figure 4 evidences a good degree of transfection occurring at different incubation times. The nanoplexes induced a significant shift in the fluorescence peak of SKMM1 cells after 24 h, which was more relevant after 48 h. This finding was probably related to the efficient internalization of the nanoplexes, which allowed an increase in the time-dependent fluorescence as a consequence of the controlled release of the probe.

### *In vivo* antitumor efficacy of nanoplexes

**Figure 5:**
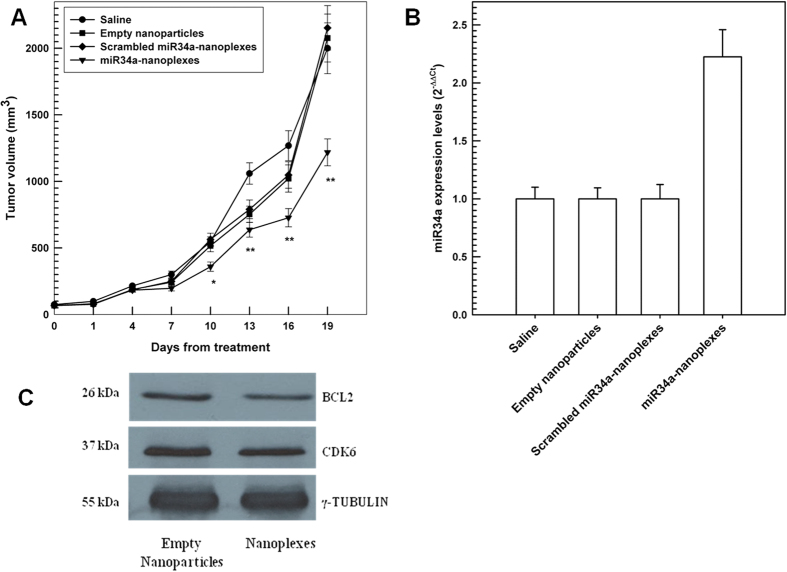
Panel A: *In vivo* antitumoral activity of miR-34a-loaded chitosan/PLGA nanospheres in SKMM1 xenografts by tail vein injections. Immunodeficient NOD–SCID mice were injected in the interscapular area (sc) with 1 × 10^6^ MM cells suspended in phosphate buffered saline. When tumor volume reached approximately 20 mm^3^ as measured by a caliper, the animals were randomly separated into 8 groups (5 animals in each group) and treated systemically (*tail vein*) once every three days for a total of six administrations with the following formulations: saline solution (NaCl 0.9% w/v) as the control, miR-34a-loaded nanoplexes, scrambled miR-34a-loaded nanoplexes and empty nanoplexes. Each animal received a dosage of 20 μg of miR-34a. The tumor volume was measured with an electronic caliper every 3 days and the resulting values represent the average tumor volume of each group ± SD (*P<0.05, **P<0.01). Panel B. q-RT-PCR analysis of miR-34a using total RNA from tumors of mice treated with the different formulations by tail vein administration. Raw Ct values were normalized to RNU44 and expressed as ΔCt values calculated with respect to miR-34a levels in tumors, using the comparative cross threshold method. Values are the mean of four different experiment ± SD. Panel C: western blotting of BCL2 and CDK6 protein in MM tumors of mice treated with miR34a-loaded nanospheres versus its scrambled sequence-loaded nanospheres by tail vein administration. The protein loading control was γ-tubulin. Experiments were performed in triplicate

The chitosan/PLGA nanospheres must not only ensure the protection of the entrapped genetic material from serum enzymes but also promote its localization into specific cells/tissues in order to carry out its therapeutic action. The *in vivo* antitumor efficacy of miR34a-loaded nanoplexes was investigated on SKMM1 xenograft models in SCID mice. After 5 injections, a remarkable decrease in tumor growth was obtained with respect to the control group (p < *0.01*) (Fig. 5, panel A). This result is particularly relevant because of the poor vascularization of the interscapular area. Moreover, it confirmed the antitumor effect of miR-34a because the other formulations, such as the empty nanoplexes and the nanoplexes containing the scramble sequence, had no effect on the proliferation of the tumor masses. Moreover, the mice treated with miR-34a-nanoplexes evidenced a higher survival rate after the last administration (see supplementary information). These findings evidenced the suitable bioavailability of the chitosan/PLGA nanoplexes, which were able to assure the localization of the miR-34a in tumor areas.

However, it should also be considered that the positive charge of the nanoplexes can have a negative effect on their biopharmaceutical features, because the mononuclear phagocyte system recognizes cationic systems, thus potentially inhibiting the accumulation of the nanoplexes in the target sites. Moreover, a certain amount of miR-34a remained adsorbed on the colloidal surface, being therefore degraded by serum enzymes. These aspects could be modulated in order to improve the therapeutic efficacy of the miR-34a-loaded nanoplexes.

A quantitative expression of miR-34a levels in retrieved tumors was analyzed by RT-PCR (Fig. 5, panel B). As previously described, miR-34a is a tumor suppressor which is down-regulated in several types of cancer, including MM, and its mechanism appears to be related to the direct modulation of certain targets such as Bcl-2 and CDK6. The evaluation of miR-34a expression in retrieved tumor masses treated with miR-34a-loaded nanoplexes evidenced a **~**2-fold increase in the compound concentration as compared to the other formulations. This result evidenced the effective intracellular delivery of miR-34a and its efficient avoidance of the lysosomes, probably thanks to the proton-sponge effect promoted by chitosan^18^. Moreover, the down-expression of both Bcl-2 and CDK6 confirmed the effective increase of the levels of miR-34a in the tumors (Fig. 5, panel C).

**Figure 6:**
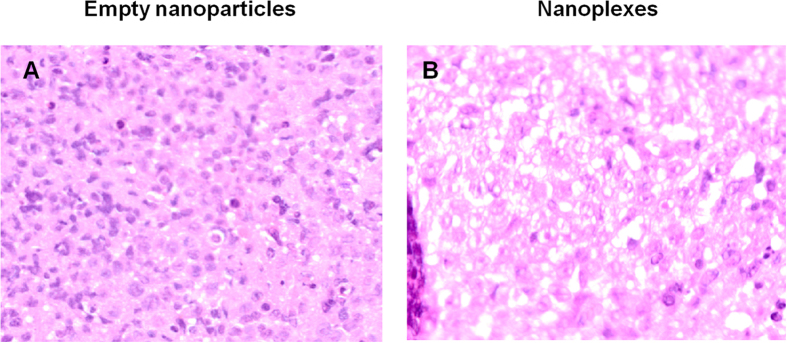
Histological analysis (100 × magnification) of neoplastic masses excised from immunodeficient NOD-SCID mice bearing SKMM1 human multiple myeloma xenograft tumors at the end of the experiments. Samples 7–10 μm thick were sliced and stained using the eosin B/hematoxylin method.

The histological analysis of the retrieved tumor masses evidenced the appearance of large apoptotic and necrotic areas in the mice treated with the nanoplexes, while the empty nanosystems did not induce any degree of toxicity (Fig. 6). The safe feature of the miR-34a nanoplexes was evidenced by the analysis of the other organs which showed no sign of tissue toxicity, hence confirming the specific activity of miR-34a against tumor tissue (see supplementary information).

All together these findings demonstrate that the miR-34a-loaded nanoplexes could efficiently exert their anticancer effect following *in vivo* systemic administration.

## Discussion

Nowadays nanomedicine is a fundamental therapeutic tool and a promising option that needs to be thoroughly investigated in order to come up with efficient treatments for many types of malignancies^19^,^20^. In fact, since the mid-’90 s with the development of liposomal doxorubicin, many colloidal formulations have been realized and put to use in clinical practice^21^. The modulation of the biopharmaceutical properties of the “old drugs” through their encapsulation in innovative drug delivery systems makes them more effective at lower dosages and results in decreased side effects.

Despite a number of advancements in pre-clinical and clinical practice^22^,^23^,^24^, MM remains an incurable disease. One of the most important therapeutic options presently under investigation are miRNAs. The promising results obtained in the treatment of different cancers need to be correlated with the formulation used to deliver genetic materials. In fact, the use of biocompatible compounds able to prevent toxicity and to be modulated in order to easily modify the biopharmaceutical properties of the entrapped drug is a fundamental requisite for reasonable application in clinical practice. A point of strength of this investigation regarding the preparation of a nanomedicine for the efficacious delivery of miR-34a, is the use of fitting materials such as PLGA and chitosan, which have already been approved by the FDA and the EMA for pharmaceutical application^25^,^26^. Because of the cationic features of the nanoplexes, an efficacious anticancer effect against MM was provided by this nanomedicine by way of the intracellular localization of miR-34a as demonstrated by the *in vitro* and *in vivo* tests. With the present we provide the first report regarding the development of safe colloidal polymeric nanosystems able to efficiently deliver the compound, targeting it against MM cells, and avoiding the appearance of toxicity in healthy tissues. The co-encapsulation of miR-34a and “old drugs” in the described nanosystems could have an enormous anticancer effect against MM^27^,^28^.

Further advancement of this nanomedicine could be made using a bio-conjugational approach, employing targeting moieties in such a way as to bring about the selective delivery of the encapsulated drug compound towards the site of action, thus achieving an even more active nanomedicine against MM. The approach herein reported could be a solid starting point for the potential translation of miRNA-based therapy into clinical practice, in the battle against MM and other aggressive types of cancer.

## Methods

### Materials

Poly(D,L-lactide-co-glycolide) (PLGA) 75:25 (molecular weight 66.000–107.000 Da), 3-[4,5- dimethyl thiazol-2-yl]-3,5-diphenyltetrazolium bromide salt (used for MTT-tests), dimethyl sulfoxide (DMSO), amphotericin B solution (250 μg/ml), phosphate saline tablets (for the preparation of phosphate buffer solution pH **~**7.4), sodium dodecyl sulfate (SDS), agarose, ponceaus solution and ethidium bromide were purchased from Sigma Chemicals Co. (Milan, Italy). Chitosan (low molecular weight, <10.000 Da, copolymer of β(1 → 4) linked 2-acetamido-2-deoxy-β-D-glucopyranose and 2-amino-2-deoxy-β-D-glucopyranose, degree of deacetylation 85%) was provided by Acef S.p.a. (Fiorenzuola D’Arda, Piacenza, Italy). Poloxamer 188 (Pluronic^®^ PE 6800 or Pluronic^®^ F68) was purchased from BASF (Aktiengesellschaft, Ludwigshafen, Germany). Human multiple myeloma cell lines (SKMM1 and RPMI8226) were provided by the AIRC 5 × 1000 research network. RPMI-1640 culture media, enriched with glutamine, fetal bovine serum (FBS), trypsin-EDTA (1 × ) and penicillin–streptomycin (100 UI/mL) solution were obtained from GIBCO (Life Technologies, Monza, Italy). MiR-34a, scrambled miR-34a, FAM™ dye-labeled pre-miR negative control, ribonuclease A (RNase A), Oligofectamine^TM^ transfection reagent, TRIzol^®^ reagent, NP40 lysis buffer, TaqMan^®^ microRNA assay, RNA*later*^®^ solution were furnished by Life Technologies (Monza, Italy). dsRNA ladder was purchased from New England BioLabs^®^ inc. (Massachusetts, USA). Anti-Bcl2 (sc-492) and anti-γ-tubulin (D-10) were purchased from Santa Cruz Biotechnology, inc. (Dallas, Texas, USA). Anti-CDK6 (DSC83) was obtained from Cell Signaling Technology^®^ (Danvers, Massachusetts). Quick Start^TM^ bradford protein assay and blotting-grade blocker were purchased from Bio-Rad (Hercules, California). All other materials and solvents used throughout this investigation were of analytical grade (Carlo Erba, Milan, Italy).

### Preparation of nanospheres

Chitosan/PLGA nanospheres were prepared following the nanoprecipitation method of the pre-formed polymer in an aqueous solution as previously described^15^. Briefly, PLGA (6 mg) was dissolved in 2 ml of acetone at room temperature and added to an acidified aqueous phase (5 ml) made up of MilliQ water (acetic acid 0.5% v/v) containing chitosan (0.3 mg) and poloxamer 188 (1% w/v) which had been homogenized by an ultraturrax (Ultraturrax T25, IKA^**®**^ Werke) at 24000 rpm for 1 min and then mechanically stirred at 600 rpm for 3 h with the aim of allowing the evaporation of the organic solvent (Table 1). The aqueous phase was previously filtered using a 0.45 μm polyamide filter (Chromafil^®^ Xtra PA-45/25, Macherey Nagel, Germany) in order to avoid the formation of chitosan aggregates. Different amounts of miR-34a were added to the aqueous solutions (100–500 μg) before homogenization in order to investigate entrapment efficiency (Table 1). The formulations were purified by centrifugation (70,000 rpm, 4 °C for 1 h, Optima TL Ultracentrifuge, Beckman).

### Physicochemical characterization of nanoparticles

Mean sizes, size distribution and surface charges were evaluated using photon correlation spectroscopy (PCS), (Zetasizer Nano ZS, Malvern Instruments Ltd., Spring Lane South, Worchester Shine, England) as previously reported^29^. Results were expressed as the mean ± standard deviation. The morphology of the nanospheres was evaluated using a transmission electron microscope (Philips, Eindhoven, The Netherlands). Samples were analyzed at 100 kV. A drop of the sample was deposited on a copper screen coated with carbon. The sample was dried and then contrasted with uranyl acetate for 2 min and then washed with distilled water.

The stability of the polymeric nanoparticles was determined by Turbiscan Lab^®^ Expert analysis (Formulaction, L’Union, France)^30^ (see supplementary information).

### Interaction of miR-34a and nanospheres

The binding of miR-34a with nanoparticles was qualitatively determined by agarose gel electrophoresis. Briefly, nanoplexes were centrifuged and the pellet destroyed with an aqueous solution of SDS (1.0% w/v) in order to assay the presence of miR-34a. To each 10 μl sample, 2 μl of 6 × loading dye were added totaling a final volume of 12 μl. The complexes were then loaded onto 1% agarose gel in Tris-acetate-EDTA (TAE) buffer containing 0.5 μg/μl of ethidium bromide. Electrophoresis was run at 120 V for 30 min. The miR-34a bands were visualized by using a UV-20 transilluminator (Hoefer Pharmacia Biotech Inc., San Francisco, CA, USA). Naked miR-34a and blank nanoparticles were used as the controls.

### Evaluation of entrapment efficiency

The amount of miR-34a encapsulated in the nanoplexes was spectrophotometrically determined. The nanoplexes were centrifuged and the supernatants analyzed by means of a NanoDrop^®^ ND1000 spectrophotometer at the wavelength of 260 nm. RNA integrity was evaluated as the A_260_/A_280_ ratio. A value > 1.8 was an index of suitable purity and integrity of miR-34a.

### Evaluation of serum stability of nanoplexes

Chitosan/PLGA nanoparticles, nanoplexes and miR-34a/Oligofectamine^TM^ were incubated in 60% (v/v) FBS in order to investigate size modification as a function of time^31^. Briefly, 200 μl of the different formulations were added to 1 ml of medium and incubated at 37 °C for different incubation times at 600 rpm. The size of the nanosystems was evaluated through DLS as previously described, following a 1:50 dilution of samples.

### RNase potection assay

The ability of chitosan/PLGA nanoplexes to protect miR-34a against degradation was evaluated through agarose gel electrophoresis. Briefly, 0.3 μl of Ribonuclease A (RNase A) (2 units) were added to 4 μl of nanoplexes or 0.2 μg of naked miRNA. Samples were incubated at 37 °C for 1 h. Successively, all samples were incubated with 4 μl of EDTA (0.25 M) for 10 min, in order to disactivate the RNase A, and then mixed with 1.0% sodium dodecyl sulfate (SDS), previously dissolved in 1 M NaOH, arriving at the final volume of 18 μl. The final samples were incubated for 1 h and gel electrophoresis was carried out in 1% (w/v) agarose gel for 40 min at 120 V using TAE buffer. The control was naked miR-34a. The experiments were also carried out as a function of time ranging from 1 h to 48 h.

### Cell cultures

Multiple Myeloma (MM), RPMI8226 and SKMM1 cells, were maintained in suspension in tissue culture treated flasks (75 cm^2^) in RPMI 1640. All media were supplemented with 10% (v/v) FBS, 100 μg/ml streptomycin, 100 IU/ml penicillin, and 250 μg/ml amphotericin B and then incubated at 37 °C in a humidified atmosphere with 5% CO_2_ (Forma^®^ Series II Water-Jacketed CO_2_ Incubator, Thermo Scientific, Germany). Fresh medium was substituted every 48 h.

### *In vitro* cytotoxic activity of nanoparticles

The 3-[4,5-dimethylthiazol-2-yl]-3,5-diphenyltetra-zolium bromide dye (MTT) test was performed on the MM cell lines in order to investigate the cytotoxicity of the colloidal formulation as a function of both PLGA or miR-34a concentration and incubation time (see supplementary information).

The cultured cells were plated in 96-well culture dishes (2 × 10^4^ cells/0.2 ml), incubated for 24 and 48 h at 37 °C in a humidified atmosphere of 5% CO_2_ and then treated with different concentrations of empty or miR-34a-loaded nanoplexes. Results were the mean of six different experiments ± standard deviation. The untreated cells were used as the control.

### *In vitro* transfection studies

The transfection efficiency of nanospheres was performed using a FAM^TM^-labeled pre-miR. A complex of FAM^TM^-labeled pre-miR/Oligofectamine^TM^ was used as positive transfection control while untreated cells were used as the negative control. The complex was obtained according to the manufacturer’s instructions (see supplementary information).

### Guidelines of animal protocols

Immunodeficient NOD–SCID mice (Harlan Laboratories, Inc., Indianapolis, 4–6 weeks old) were housed and monitored in our animal research facility. All experimental procedures and protocols had been approved by the Institutional Ethical Committee (“Magna Graecia” University) and performed according to protocols approved by the National Directorate of Veterinary Services (Italy, Permit Number: 235 on June 30^th^, 2011). In accordance with institutional guidelines, the mice were sacrificed when their tumors reached 2 cm in diameter or in the event of paralysis or major compromise of their quality of life, in order to prevent unnecessary suffering.

### *In vivo* efficacy of miR-34a-loaded nanoparticles

Immunodeficient NOD–SCID mice were injected in the interscapular area (sc) with 1 × 10^6^ MM cells suspended in phosphate buffered saline. When tumor volume reached approximately 20 mm^3^ as measured by a caliper, the animals were randomly separated into 8 groups (5 animals in each group) and treated systemically (*tail vein*) once every three days for a total of six administrations with the following formulations: saline solution (NaCl 0.9% w/v) as the control, miR-34a-loaded nanoplexes, scrambled miR-34a-loaded nanoplexes and empty nanoplexes. Each animal received a dose of 20 μg of miR-34a. Body weight, feeding behavior and motor activity of the mice were evaluated as indicators of general health. Four groups were sacrificed three days after the last administration, while the other four groups were monitored till death, thus investigating the survival rate of treated mice. The volume of the tumor masses was calculated according to the following equation:


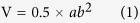


where *a* and *b* were the long and short diameters of the tumor, respectively. Tumors were collected and placed in RNA*later*^®^ for RNA isolation.

For the histological analysis, at the end of the experiment tumors and organs were immediately immersed in 4% (w/v) buffered formaldehyde and after 24 h samples were washed, dehydrated and embedded in paraffin. Haematoxylin-eosin staining was carried out by cutting 2 mm sample slices, which were mounted and stained on poly-lysine slides. Samples were observed by light microscopy using a Nikon i55 (Nikon Corporation, Tokyo, Japan) optical microscope.

### Quantitative real-time amplification of miRNA

Total RNA was extracted from the tumors of mice treated with miR-34a-loaded nanoplexes using TRIzol^®^ Reagent (Invitrogen), in compliance with the manufacturer’s instructions. Tissue disruption was carried out using a Tissue Ruptor^®^ (Qiagen, Venlo, Netherlands) according to the manufacturer’s instructions. Total RNA was reverse-transcripted into oligo-dT-primed cDNA using the High Capacity cDNA Reverse Transcription Kit (Applied Biosystems). The single-tube TaqMan miRNA assays were used to detect and quantify mature miR-34a according to the manufacturer’s instructions by the use of the StepOne Thermocycler and the sequence detection system (Applied Biosystems). The miR-34a was normalized on RNU44 (Ambion). An oligonucleotide (Life Technologies) with a scrambled sequence was used as a miR-negative control. Comparative real-time polymerase chain reaction (RT-PCR) was performed in triplicate, including no-template controls. Relative expression was calculated using the comparative cross-threshold (Ct) method^32^.

### Western blotting analysis

Total protein from tumors of mice treated with miR-34a-loaded nanoplexes was prepared with the NP 40 reagent supplied with phosphatase and protease inhibitors. 1 ml of NP40 was added to the tumors and homogenized by Tissue Ruptor^®^ (Qiagen, Venlo, Netherlands) according to manufacturer’s instructions. Proteins were separated using SDS-Page. Anti-Bcl2 and anti-CDK6 antibodies were used during experiments. Visualization of immune complexes was carried out by chemiluminescence using the ECL-Plus kit (GE-Healthcare). The images of the western blots were obtained by computer-scanning the electronic image and processed using Adobe Photoshop CS installed on a Power Macintosh G5. Each experiment was performed in triplicate.

### Statistical Analysis

One-way ANOVA was used for the statistical analysis of the various experiments. A posteriori Bonferroni t-test was carried out to check the ANOVA test. A p value of <0.05 was considered statistically significant. Values are reported as the average mean ± standard deviation.

## Additional Information

**How to cite this article**: Cosco, D. *et al.* Delivery of miR-34a by chitosan/PLGA nanoplexes for the anticancer treatment of multiple myeloma. *Sci. Rep.* **5**, 17579; doi: 10.1038/srep17579 (2015).

## Supplementary Material

Supplementary Information

## Figures and Tables

**Table 1:** Physico-chemical parameters of nanoparticles prepared both in the absence and in the presence of pre-miR-34a.^a^

Sample	miR-34a (μg)	Mean size (nm)	PI^b^	Zeta potential (mV)	EE (%)^c^
A	-	176.6 ± 1.9	0.21 ± 0.03	45.1 ± 1.5	–
B	100	178.0 ± 2.2	0.19 ± 0.03	40.3 ± 2.0	95.1 ± 2.7
C	200	170.3 ± 0.4	0.19 ± 0.01	37.5 ± 1.1	93.2 ± 3.1
D	300	165.2 ± 1.5	0.18 ± 0.01	32.7 ± 0.2	85.5 ± 3.3
E	400	154.3 ± 2.5	0.19 ± 0.02	30.9 ± 1.7	62.3 ± 4.1
F	500	150.7 ± 1.8	0.16 ± 0.03	25.1 ± 1.6	49.1 ± 2.1

^a^Each value represents the average of three different experiments ± standard deviation.

^b^Polidispersity index.

^c^Entrapment efficiency.
