# Supplementary material for: Delivery of miR-34a by chitosan/PLGA nanoplexes for the anticancer treatment of multiple myeloma
Source: Sci Rep. 2015 Dec 1;5:17579. doi: 10.1038/srep17579 (PMC4665167; doi:10.1038/srep17579)
Supplement: Supplementary Information [file srep17579-s1.pdf]

Delivery of miR-34a by chitosan/PLGA nanoplexes for anticancer treatment of multiple myeloma

Donato Cosco[1,4], Felisa Cilurzo[1], Jessica Maiuolo[1], Cinzia Federico[2], Maria Teresa Di Martino[2], Maria Chiara Cristiano[1], Pierfrancesco Tassone[2,3], Massimo Fresta[1,4*], Donatella Paolino[2,4*]

[1]Department of Health Sciences, University "Magna Græcia" of Catanzaro, Campus Universitario "S. Venuta", Viale S. Venuta, Germaneto, I-88100 Catanzaro, Italy.
[2]Department of Experimental and Clinical Medicine, University "Magna Græcia" of Catanzaro, Campus Universitario "S. Venuta", Viale S. Venuta, Germaneto, I-88100 Catanzaro, Italy.
[3]Sbarro Institute for Cancer Research and Molecular Medicine, Center for Biotechnology, College of Science and Technology, Temple University, Philadelphia, PA, US.
[4]IRC FSH- Interregional Research Center for Food Safety & Health, University of Catanzaro "Magna Græcia", Campus Universitario "S. Venuta" - Building of BioSciences, Viale S. Venuta, I-88100 Germaneto - Catanzaro, Italy.

*Corresponding Authors
E-mail: fresta@unicz.it ; phone +39 0961 369 4118; Fax +39 0961 369 4237
E-mail: paolino@unicz.it; phone +39 0961 369 4221; Fax +39 0961 369 4237

SUPPLEMENTARY INFORMATION

Materials and methods

Physico-chemical characterization of nanoparticles

Photon correlation spectroscopy was used to evaluate mean sizes, z-potential and polydispersity index of both PLGA/chitosan nanoparticles and nanoplexes. A Zetasizer Nano ZS (Malvern Instruments Ltd., Worchestershire, UK) was used for these experiments. A third-order cumulant fitting correlation function was applied. The real and imaginary refractive indices were set at 1.59 and 0.0, respectively. The medium refractive index (1.330), medium viscosity (1.0 mPa × s) and the dielectric constant (80.4) were set before the experiments began. Samples were analyzed using quartz cuvettes. Instrument calibration was carried out using a monodisperse polystyrene latex (126 nm) as a standard sample.

The same instrument was used to determine z-potential values and a Smoluchowsky constant F (Ka) of 1.5 was applied to calculate the z-potential values from the electrophoretic mobility of the nanosystems. The various measurements were carried out in triplicate on three different batches (ten determinations for each batch). Results were the mean of three different experiments ± standard deviation.

Stability of nanoparticles by Turbiscan Lab® Expert

The stability of nanoplexes was evaluated using a Turbiscan Lab® Expert apparatus following the variation of their back-scattering (ΔBS) profiles as a function of time. The following equation was applied:

$$\text{Eq. 1} \quad BS = 1/\sqrt{\lambda^*}$$

where λ* was the mean distance covered by the protons in the analyzed dispersion. From the physical point of view, the $\lambda^*(\phi,d)$ value in the analyzed dispersion was evaluated by using the following equation:

$$\text{Eq. 2} \quad \lambda^*(\Phi, d) = [(2d)/(3\Phi(1-\mathrm{g})\mathrm{Qs})]$$

where ϕ is the volume fraction of particles, d is the mean diameter of particles and $g(d)$ and $Qs\ (d)$ are the optical parameters given by the Mie theory. The obtained *BS* data were then elaborated as ΔBS profiles by the Turbiscan EasySoft Converter.

The measurements were carried out using a pulsed near infrared LED at a wavelength of 880 nm for 1 h. Two different synchronous optical sensors received the light transmitted and backscattered by the samples at an angle of 180° and 45°, respectively, with respect to the incident radiation. The two sensors scanned the entire height (8 mm) of the various samples (6 ml) for 1 h. Experimental data were correlated in percentage to the light flux of two reference standards: a polystyrene latex suspension (absence of transmission and maximum backscattering) and a silicon oil (maximum transmission and absence of backscattering). This analysis is based on the variation of the particle volume fraction (migration) and mean size, both of which are strictly related to the backscattering and transmission signals. The migration of the systems from the bottom of the cell to the top represents a decrease in the concentration at the bottom of the sample which, in turn, denotes a decrease in the backscattering signal (negative peak) and an increase in the intensity of the transmission (positive peak). The opposite is true at the top of the sample. A variation of the backscattering profile within the interval ±5% evidences suitable stability of the formulation.

Evaluation of cytotoxic activity

The cultured cells were plated in 96-well culture dishes (2×10^4 cells/0.2 ml) and incubated for 24 h at 37 °C. The culture medium was then removed, replaced with the different formulations and incubated for 24 h and 48 h. Every plate had 8 wells with untreated cells as the control and 8 wells with cells treated with empty PLGA/chitosan nanoparticles. After each incubation period, 20 µl of tetrazolium salt solubilized in PBS solution (5 mg/ml) were added to every well and the plates were incubated again for 3 h. The medium was removed and the formazan salts (precipitated on the well bottom after oxidation) were dissolved with 200 µl of a mixture of DMSO/ethanol (1:1 v/v), by shaking the plates for 20 min at 230 rpm (IKA® KS 130 Control, IKA® WERKE GMBH & Co, Staufen, Germany). The solubilized formazan was quantified with a microplate spectrophotometer (Multiskan MS 6.0, Labsystems) at a wavelength of 540 nm, with reference at a wavelength of 690 nm. The percentage of cell viability was calculated according to the following equation:

$$\text{Eq. 3} \quad cell\ viability(\%) = AbsT/AbsC\times100$$

where *AbsT* is the absorbance of treated cells and *AbsC* is the absorbance of control cells. The formazan concentration is directly proportional to the cell viability, which was reported as the mean of six different experiments ± SD.

In vitro transfection studies

MM cells were seeded in 6-well culture dishes (1×10^6 cells/well) in an antibiotic-free medium containing the formulations (3 pmol of FAM™-labeled pre-miR/well). After 6 h incubation, the cells were washed and incubated for 24 and 48 h with fresh medium. Successively, the cells were centrifuged, the obtained supernatant was removed and the pellet was re-suspended in pre-chilled PBS. The cells were then transferred into a 5 ml polystyrene round-bottom tube for subsequent flow-cytometry analysis. The fluorescence intensities of FAM were recorded in the FL1 channel using a FACSCan (Becton Dickinson, USA) flow cytometer. The experiments were performed in triplicate.

Serum incubation

PLGA/chitosan nanoparticles, nanoplexes and Oligofectamine/miR-34a complex were incubated in 60% FBS in order to investigate size modification[1]. Briefly, 200 µl of the different formulations were added to 1 ml of 60% FBS and incubated at 37 °C for 48 h while stirring at 600 rpm. The size of the nanosystems was evaluated by DLS as previously described after a 1:50 dilution of the samples.

Results and Discussion

The ability of the nanoparticles to interact and retain miR-34a and the effective encapsulation of this compound were qualitatively investigated through agarose gel electrophoresis. The characteristic fluorescent band confirmed that the structure of the nucleic acid was not destabilized during the preparation procedure (Figure S1).

The stability of the various colloidal formulations was evaluated by Turbiscan Lab® Expert. Namely, variations greater than 5% either as a positive or as a negative value in the graphical scale of backscattering or transmission pathways are representative of an unstable colloidal formulation. The suitable stability of empty PLGA/chitosan nanoparticles is shown in Figure S2, panel A; for instance the *ΔBS* and *ΔT* profiles are both on the base-line thus evidencing no instability phenomenon. The ability of these systems to efficiently entrap the drugs while remaining stable is evidenced in Figure S1, panel B. In particular, it is possible to observe the *ΔBS* and *ΔT* profiles of the nanoplexes prepared with 300 µg of miR-34a and it is possible to appreciate a slight variation in the transmittance with respect to the unloaded systems. Moreover, the evaluation of the stability kinetic of the two formulations confirmed their high degree of stability, showing the overlapping of the *ΔBS* profiles (Figure S3).

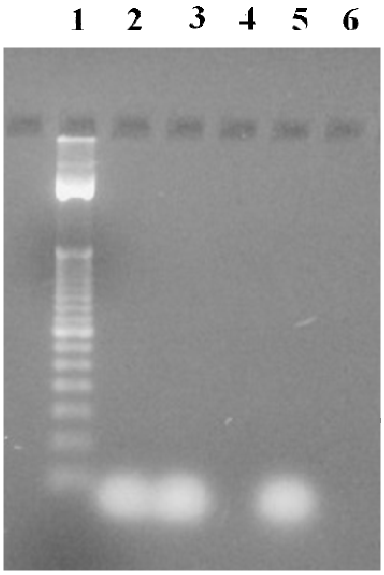

1: ladder
2: miR-34a
3: Nanoplexes
4: Empty nanoparticles
5: Pellet of nanoplexes
6: Supernatant of nanoplexes

Figure S1. Gel retardation assay. The nanoplexes were centrifuged and the pellet destroyed with an aqueous solution of SDS (1.0 % w/v) in order to assay the presence of miR-34a. To each 10 µl sample, 2 µl of 6× loading dye were added totaling a final volume of 12 µl. The complexes were then loaded onto 1% agarose gel in Tris-acetate-EDTA (TAE) buffer containing 0.5 µg/µl of ethidium bromide. Electrophoresis was run at 120 V for 30 min. The miR-34a bands were visualized using a UV-20 transilluminator (Hoefer Pharmacia Biotech Inc., San Francisco, CA, USA). Naked miR-34a and blank nanoparticles were used as the controls.

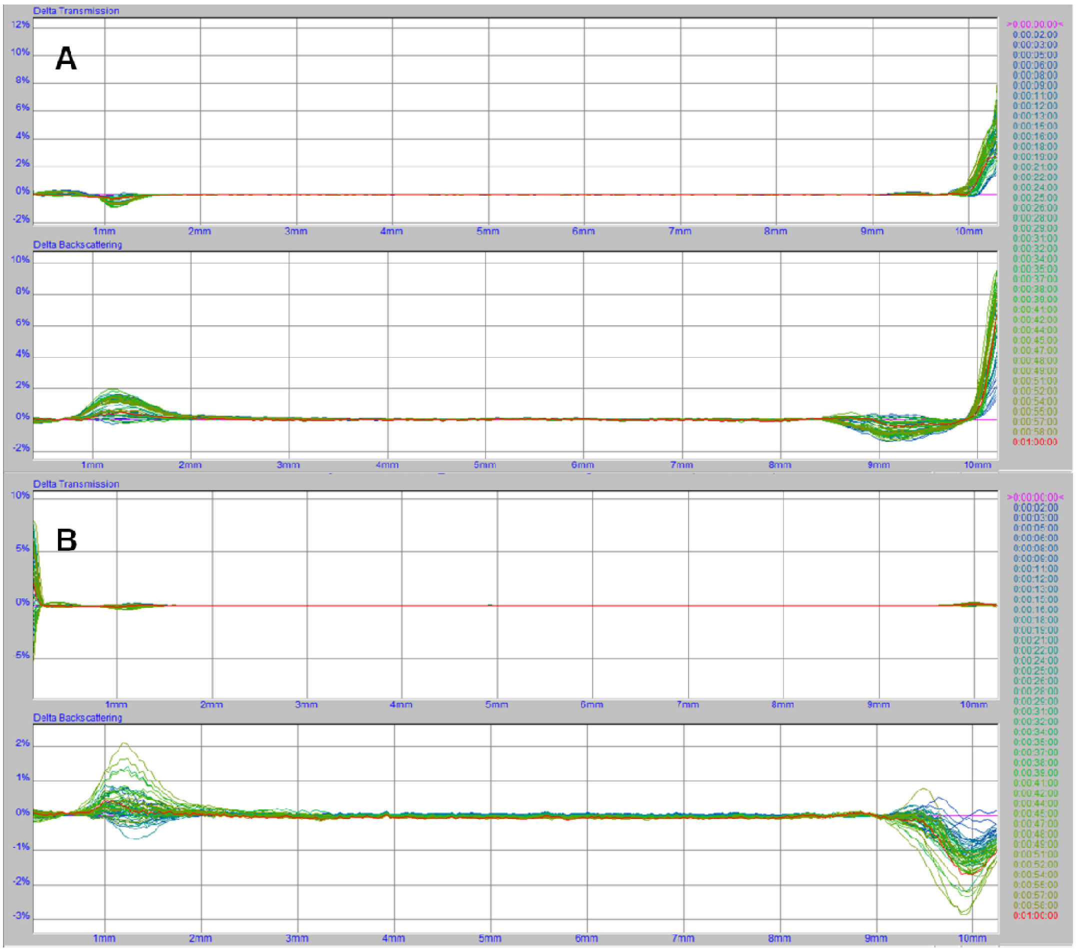

Figure S2. Transmission and backscattering profiles of empty PLGA/chitosan nanoparticles (*A*) and nanoplexes prepared with 300 µg of miR-34a (B) using Turbiscan Lab® Expert. Data are reported as a function of time (0–1 h) and sample height (from 2 to 9 mm).

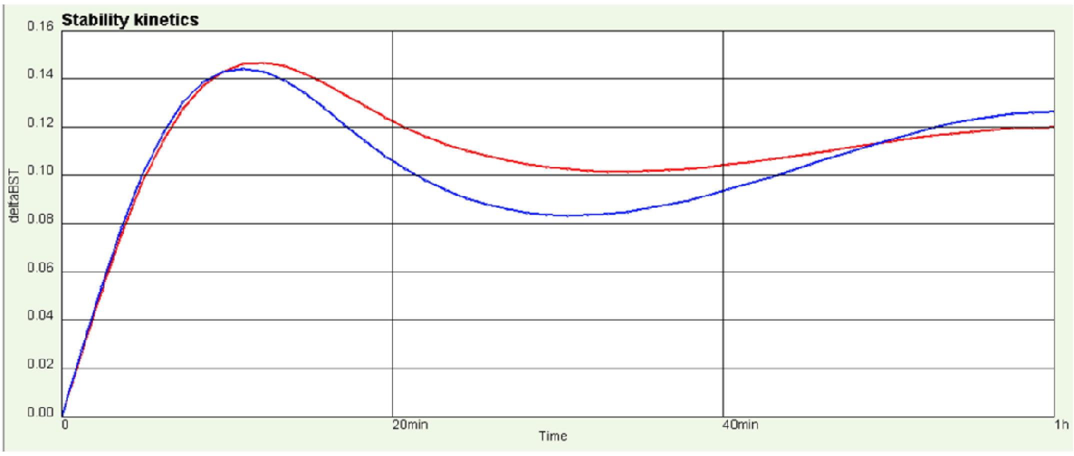

Figure S3. Kinetic stability profiles of empty PLGA/chitosan nanoparticles (red) and nanoplexes prepared with 300 µg of miR-34a (blue) by using Turbiscan Lab® Expert. Data are reported as a function of time (0–1 h).

The stability of nanosystems in 60% FBS was also investigated in order to predict the *in vivo* behavior of the formulations. Figure S4 evidences a proportional time-dependent increase of the mean sizes of empty nanoparticles and nanoplexes but shows that it is significantly less than that of the oligofectamine/miR-34a complex. This confirmed the greater effectiveness of the PLGA/chitosan nanoparticles as novel gene delivery systems with respect to the traditional formulations to be used for the *in vivo* experiments.

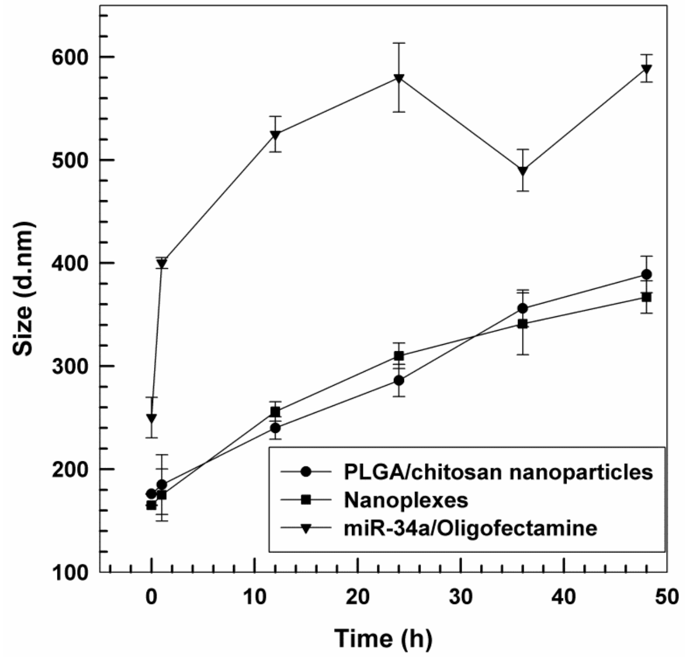

Figure S4. Serum stability of different formulations obtained through the incubation technique as a function of time. Nanoplexes and oligofectamine/miR-34a complex were prepared using 300 μg of miRNA.

The systemic administration of miR-34a-loaded PLGA/chitosan nanoparticles favored a better survival rate of mice and did not induce any degree of toxicity to organs with respect to the empty formulations, confirming both the great biocompatibility of the components of the nanosystems and the specific antitumor action of the genetic material against MM cells (Figures S5 and S6).

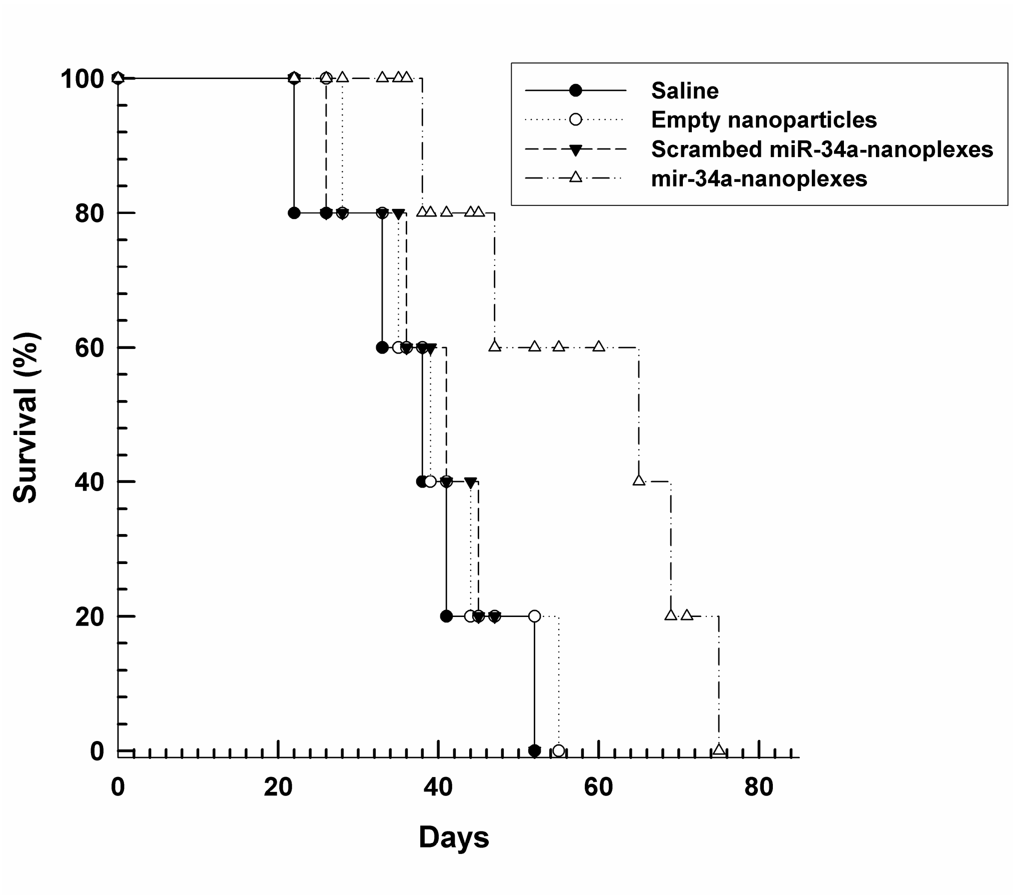

Figure S5. Survival rate of NOD-SCID mice bearing SKMM1 xenografts treated with the different formulations after the last administration (at day 18).

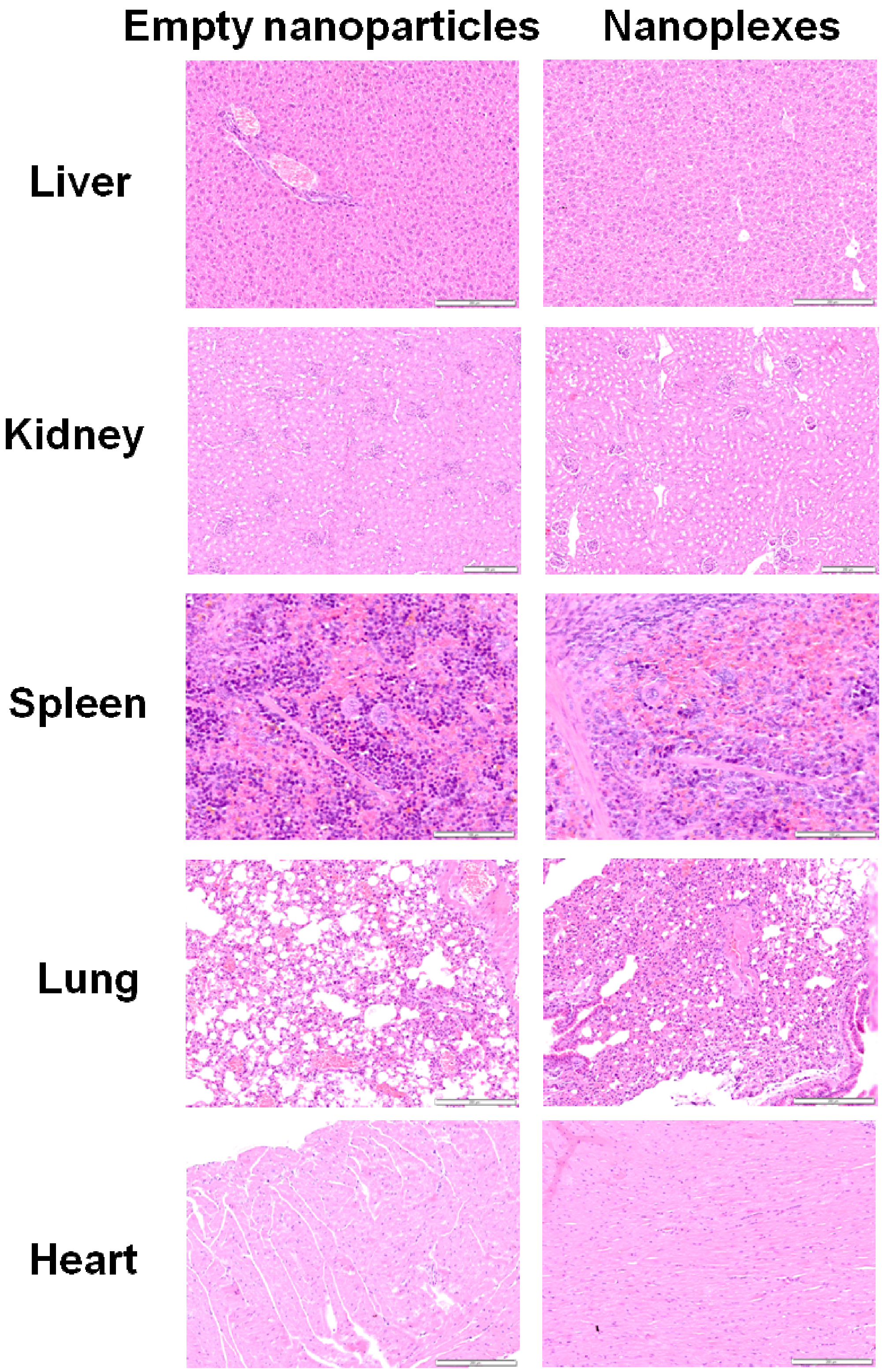

Figure S6. Histological analysis of different organs excised from immunodeficient NOD-SCID mice bearing human multiple myeloma xenograft tumors, treated with empty PLGA/chitosan nanoparticles and nanoplexes, at the end of the experiments. Samples 7–10 μm thick were sliced and stained using the eosin B/hematoxylin method.

References

1. Wolfram, J. *et al.* Shrinkage of pegylated and non-pegylated liposomes in serum. *Colloids Surf. B Biointerfaces* **114**, 294-300 (2014).
